# Barber‐say syndrome: a confirmed case of *TWIST2* gene mutation

**DOI:** 10.1002/ccr3.1014

**Published:** 2017-06-02

**Authors:** Mulakkan David Yohannan, Jennifer Hilgeman, Katlin Allsbrook

**Affiliations:** ^1^ Dayton Children's HospitalOne Children's PlazaDaytonOHUSA; ^2^ Pediatrix Medical Group of OhioOne Children's PlazaDaytonOHUSA

**Keywords:** ablepharon macrostomia syndrome, Barber‐Say syndrome, phenotype, *TWIST2* mutation

## Abstract

Barber‐Say syndrome is a rare disorder characterized by hypertrichosis, redundant skin, and facial dysmorphism. *TWIST2* gene mutation previously described in this syndrome was identified in our patient. Genetic testing is recommended in patients presenting with these phenotypic abnormalities, along with their parents, to establish de novo or inherited mutations.

## Introduction

Barber‐Say syndrome (BSS) is a rare congenital disorder characterized by hypertrichosis, redundant skin, hypoplastic or absent nipples, and dysmorphic facial features including macrostomia, bulbous nose, ocular telecanthus, eyelid ectropion, and abnormal ears 1. Patients with cleft palate and genital abnormalities have also been described 2. In 1982, Barber et al. 3 were the first to report multiple congenital abnormalities such as macrostomia, ectropion, hypertrichosis, and growth retardation in a 3‐year‐old girl. According to the literature, only 15 cases of BSS have been reported since that time 1, 2, 3, 4, 5, 6, 7, 8, 9, 10, 11, 12. Recently, it has been postulated that BSS and ablepharon macrostomia syndrome (AMS) could represent one disorder due to their similar patterns of organ involvement including skin, hair, eyes, face, and external genitalia 6. Distinguishing features include hypertrichosis in BSS only and ablepharon or microblepharon, and sparse hair in AMS.

## Case Report

Written informed consent was obtained from the patient's parents as well as IRB approval from our institution regarding publication of this case report and its accompanying images. The patient was a term infant born via spontaneous vaginal delivery to a 22‐year‐old G1P0 mother. Pregnancy was complicated by tetrahydrocannabinol (THC) use and positive group B Streptococcus status of the mother. The patient's mother had five fetal ultrasounds due to difficulty with gender identification, but no other abnormalities were detected. Apgar scores were 8 and 9 at 1 and 5 min. His birth weight was 3650 g (73rd percentile), length 51.5 cm (80th percentile), and OFC 34.2 cm (42nd percentile).

Family history was unremarkable, and consanguinity was denied. Physical examination at birth showed multiple facial dysmorphic features, and the baby was transferred to our neonatal intensive care unit for further management.

**Figure 1:**
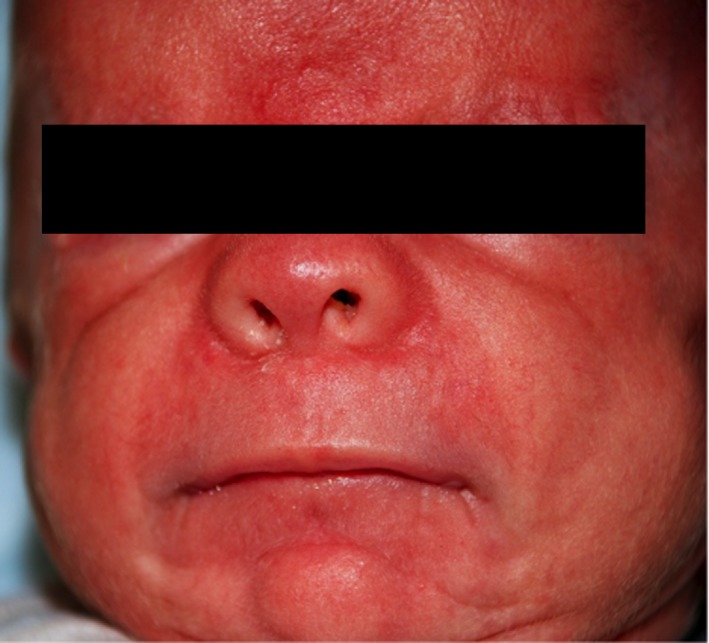
Hypertelorism, bulbous nose, macrostomia, hypoplastic eyelids, ectropion, sparse eyelashes, and absent eyebrows are notable.

**Figure 2:**
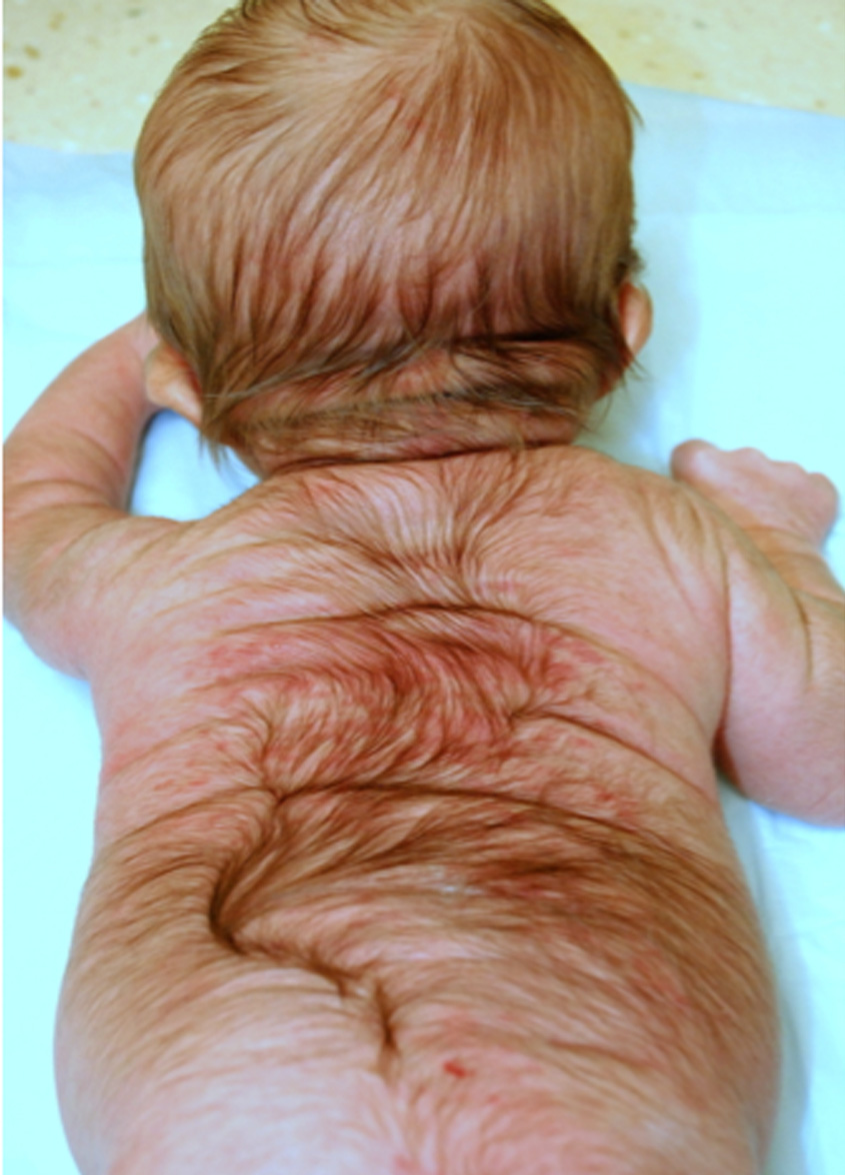
Hypertrichosis on the back.

**Figure 3:**
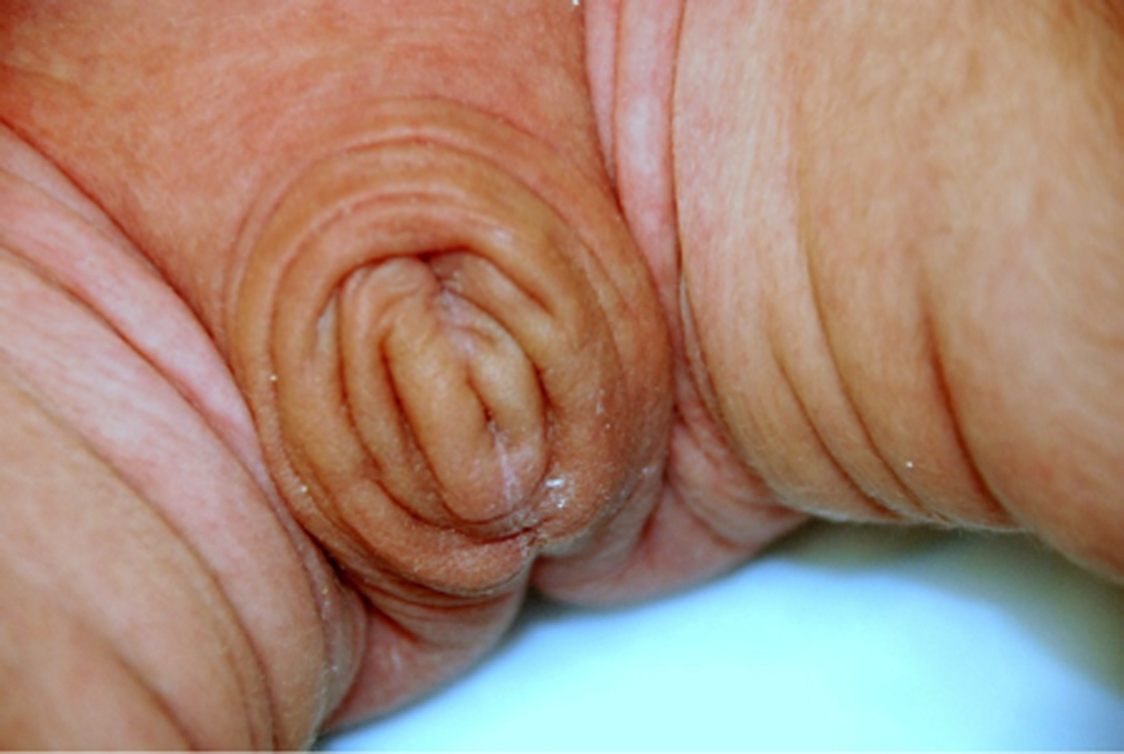
Redundant scrotal skin or “shawl scrotum”.

On admission, physical examination showed a markedly dysmorphic male with ocular hypertelorism, bulbous nose, macrostomia, hypoplastic eyelids, ectropion, sparse eyelashes, absent eyebrows, low set posteriorly rotated ears, redundant skin on the neck, and micrognathia (Fig. 1). Also observed were hypoplastic nipples, diastasis recti, and hypertrichosis on the back (Fig. 2). Patient also had a shawl scrotum concealing an anatomically normal shaft and glans of the penis (Fig. 3). Laboratory tests, echocardiogram, head MRI, voiding cystourethrogram, and hearing screen were normal. Ultrasound of the abdomen showed mild right hydronephrosis. ENT evaluation showed a soft palate cleft. Due to feeding difficulties and intermittent stridor, ENT recommended a frenulectomy and a sleep study that showed abnormal sleep architecture, obstructive sleep apnea, and micrognathia. Ophthalmology evaluation showed a normal macula, fovea, and periphery and recommended vigorous eye lubrication due to the hypoplastic eyelids and inability to close eyes. Barber‐Say syndrome was the main differential diagnosis for this patient. Marchegiani et al. 13 reported several individuals with Barber‐Say syndrome and ablepharon macrostomia who have documented pathogenic *TWIST2* mutations. *TWIST2* sequencing was ordered from Fulgent Diagnostics. Additional genetic testing for patient has not been completed to date. Genetic counseling was provided pre‐ and posttesting. Patient was discharged home on day of life eleven.

At 1 month of age, he was able to close his eyes partially, feeding and gaining weight appropriately, and had small fibromas noted on alveolar ridge and toes. Postdischarge, patient has been admitted to the hospital several times for choking episodes. At 7 months of age, patient had eye surgery with ophthalmology and plastic surgery to correct hypoplastic eyelids with skin grafting. Following this procedure, he was able to fully close the eyes. At 1 year of age, patient was meeting all developmental milestones. He continues to follow with ophthalmology, ENT for observation of cleft palate, gastroenterology for reflux, and pulmonology for obstructive sleep apnea.

## Discussion

*TWIST2* sequencing showed a pathogenic mutation: c.223G>C (p.Glu75Gln). This variant has been previously reported 13. Functional studies have shown that this mutation is in the basic helix–loop–helix DNA‐binding domain of this protein. Additionally, this mutation alters the DNA‐binding pattern of the *TWIST2* gene. This specific *TWIST2* mutation has been reported in 11 other individuals with Barber‐Say syndrome and has not been reported in any cases of ablepharon macrostomia syndrome to date 13. This mutation is in a highly conserved amino acid. *TWIST2* gene mutations were initially described in Setleis syndrome, an inherited ectodermal dysplasia disorder 14. Maternal targeted testing was performed and was normal. Paternal targeted has not been completed, as father declined genetic testing. The patient's father was phenotypically normal. Paternal testing would have helped to clarify whether this finding is paternally inherited or de novo. Additionally, it would clarify recurrence risks for the patient's family.

Many features reported in this patient overlap with reported features in other individuals with the same *TWIST2* mutation. Few male patients have been reported with a shawl scrotum 15. Haensel et al. 6 report that approximately 38 percent of males with Barber‐Say syndrome have ambiguous genitalia. Additionally, there is some phenotypic overlap in this patient between ablepharon macrostomia and Barber‐Say syndrome, including skin redundancy, macrostomia, and abnormally shaped ears and nose 6, 13. Haensel et al. 6 hypothesized that ablepharon macrostomia and Barber‐Say syndrome could represent one disorder and represent phenotypic overlap rather than a single disorder because some features are unique to both. This case demonstrates the importance of genetic testing evaluating for a *TWIST2* mutation in patients with this phenotypic presentation as well as parental testing to evaluate whether mutation was inherited or de novo. With rare disorders like Barber‐Say syndrome, it is important to document all cases so that a genotype–phenotype correlation can be made.

## Conflict of interest

There is no conflict of interest for any of the authors of this manuscript.

## Authorship

MDY: oversaw care for the patient while in the NICU as the neonatologist, photographed the patient in the case, performed the literature review, and was an integral part of the writing and editing for the manuscript; JH: participated in care for this infant in the NICU, responsible for writing the abstract, introduction, and case report, and contributed to the discussion as the corresponding author; KA: obtained genetic testing for the patient and the mother as the genetic counselor and contributed to the discussion section of the manuscript.
